# Electrocardiogram-based prediction of conduction disturbances after transcatheter aortic valve replacement with convolutional neural network

**DOI:** 10.1093/ehjdh/ztae007

**Published:** 2024-02-08

**Authors:** Yuheng Jia, Yiming Li, Gaden Luosang, Jianyong Wang, Gang Peng, Xingzhou Pu, Weili Jiang, Wenjian Li, Zhengang Zhao, Yong Peng, Yuan Feng, Jiafu Wei, Yuanning Xu, Xingbin Liu, Zhang Yi, Mao Chen

**Affiliations:** Department of Cardiology, West China Hospital, Sichuan University, No.37 Guoxue Street, Chengdu 610041, P. R. China; Department of Cardiology, West China Hospital, Sichuan University, No.37 Guoxue Street, Chengdu 610041, P. R. China; Machine Intelligence Laboratory, College of Computer Science, Sichuan University, No.24 South Section 1, Yihuan Road, Chengdu 610065, P. R. China; Department of Information Science and Technology, Tibet University, No.10 Zangda East Road, Lhasa 850000, Tibet, P. R. China; Machine Intelligence Laboratory, College of Computer Science, Sichuan University, No.24 South Section 1, Yihuan Road, Chengdu 610065, P. R. China; Department of Cardiology, West China Hospital, Sichuan University, No.37 Guoxue Street, Chengdu 610041, P. R. China; Department of Cardiology, West China Hospital, Sichuan University, No.37 Guoxue Street, Chengdu 610041, P. R. China; Machine Intelligence Laboratory, College of Computer Science, Sichuan University, No.24 South Section 1, Yihuan Road, Chengdu 610065, P. R. China; Machine Intelligence Laboratory, College of Computer Science, Sichuan University, No.24 South Section 1, Yihuan Road, Chengdu 610065, P. R. China; Department of Cardiology, West China Hospital, Sichuan University, No.37 Guoxue Street, Chengdu 610041, P. R. China; Department of Cardiology, West China Hospital, Sichuan University, No.37 Guoxue Street, Chengdu 610041, P. R. China; Department of Cardiology, West China Hospital, Sichuan University, No.37 Guoxue Street, Chengdu 610041, P. R. China; Department of Cardiology, West China Hospital, Sichuan University, No.37 Guoxue Street, Chengdu 610041, P. R. China; Department of Cardiology, West China Hospital, Sichuan University, No.37 Guoxue Street, Chengdu 610041, P. R. China; Department of Cardiology, West China Hospital, Sichuan University, No.37 Guoxue Street, Chengdu 610041, P. R. China; Machine Intelligence Laboratory, College of Computer Science, Sichuan University, No.24 South Section 1, Yihuan Road, Chengdu 610065, P. R. China; Department of Cardiology, West China Hospital, Sichuan University, No.37 Guoxue Street, Chengdu 610041, P. R. China

**Keywords:** Transcatheter aortic valve replacement, Artificial intelligence, Electrocardiogram, Conduction disturbances, Convolutional neural network

## Abstract

**Aims:**

Permanent pacemaker implantation and left bundle branch block are common complications after transcatheter aortic valve replacement (TAVR) and are associated with impaired prognosis. This study aimed to develop an artificial intelligence (AI) model for predicting conduction disturbances after TAVR using pre-procedural 12-lead electrocardiogram (ECG) images.

**Methods and results:**

We collected pre-procedural 12-lead ECGs of patients who underwent TAVR at West China Hospital between March 2016 and March 2022. A hold-out testing set comprising 20% of the sample was randomly selected. We developed an AI model using a convolutional neural network, trained it using five-fold cross-validation and tested it on the hold-out testing cohort. We also developed and validated an enhanced model that included additional clinical features. After applying exclusion criteria, we included 1354 ECGs of 718 patients in the study. The AI model predicted conduction disturbances in the hold-out testing cohort with an area under the curve (AUC) of 0.764, accuracy of 0.743, F1 score of 0.752, sensitivity of 0.876, and specificity of 0.624, based solely on pre-procedural ECG images. The performance was better than the Emory score (AUC = 0.704), as well as the logistic (AUC = 0.574) and XGBoost (AUC = 0.520) models built with previously identified high-risk ECG patterns. After adding clinical features, there was an increase in the overall performance with an AUC of 0.779, accuracy of 0.774, F1 score of 0.776, sensitivity of 0.794, and specificity of 0.752.

**Conclusion:**

Artificial intelligence–enhanced ECGs may offer better predictive value than traditionally defined high-risk ECG patterns.

## Graphical Abstract

**Graphical Abstract:**
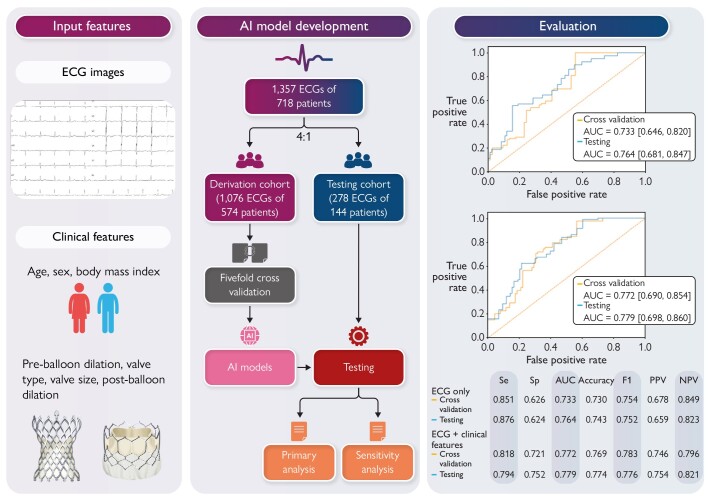
Training, validation, and evaluation of AI-enabled ECGs for the prediction of conduction disturbances after TAVR. The AI models were trained using pre-procedural 12-lead ECG images and clinical features that encompassed demographic and procedural aspects. The cross-validation set was used for training and internal validation, employing five-fold cross-validation, while a hold-out testing cohort was used for testing. The evaluation metrics employed in this study included AUC, sensitivity, specificity, accuracy, F1 score, negative predictive value, and positive predictive value.

## Introduction

Transcatheter aortic valve replacement (TAVR) is now an established and expanding treatment option for severe aortic stenosis.^1,2^ However, the high incidence of conduction disturbances after TAVR becomes a growing concern.^3–5^ Published studies have shown rates of new-onset left bundle branch block (LBBB) and permanent pacemaker implantation (PPMI) ranging from 5–65%^6^ and 2.3–37.7%,^7^ significantly higher than the benchmark rate of 3.8–5.4% in surgical aortic valve replacement.^3^

Previous studies have identified multiple predictors of conduction disturbances or PPMI after TAVR, including anatomical, electrocardiographic, and procedural factors,^8–11^ and several predictive models have been proposed.^12–15^ Regarding the electrocardiographic aspect, the most consistent predictor is the presence of right bundle branch block (RBBB), with odds ratios or relative risks ranging from 2.89 to 30.24 in different publications.^8^

Convolutional neural network (CNN)^16–18^ is a form of biologically inspired artificial intelligence (AI) that has brought dramatic changes in the pattern recognition field. It can extract subtle patterns from electrocardiogram (ECG) signals or ECG images through human-like interpretation. Artificial intelligence–powered ECGs have shown extraordinary performance in the early prediction of asymptomatic cardiac dysfunction,^19–21^ cardiomyopathy,^22,23^ arrhythmia,^24,25^ valvular disease,^26,27^ and other cardiovascular diseases.^28,29^ In particular, Raghunath *et al.*^30^ showed that AI models built with ECG traces had better performance than models built with ECG measures in predicting long-term mortality. In this context, we hypothesized that the anatomical, structural, and electrophysiological predispositions to conduction disturbances after TAVR can be captured by a properly trained neural network. To test this hypothesis, we developed an AI tool for predicting conduction disturbances after TAVR based on pre-procedural 12-lead ECGs from a single-centre retrospective TAVR cohort. Our study sought to offer valuable insights into the potential of utilizing AI-enhanced ECG to assist informed decision-making.

## Methods

### Data source and study sample

We retrospectively included patients who underwent TAVR in West China Hospital between March 2016 and March 2022. Exclusion criteria included (i) patients with prior PPMI or implanted cardiac defibrillator (ICD), (ii) patients who failed the procedure or transferred to surgical aortic valve replacement, (iii) patients who died before or during the procedure, (iv) patients without eligible pre-procedural 12-lead ECG, and (v) valve-in-valve patients. Patients were implanted with self-expandable or balloon-expandable heart valve according to the joint decision of the heart team. Four experienced operators performed TAVR collaboratively during this period in our centre (M.C., Y.P., Y.F., and J.W.). Self-expanding valve choices included VenusA and VenusA-Plus (VENUSMEDTECH, Zhejiang, China), VitaFlow (MicroPort, Shanghai), TaurusOne (Peijia, Suzhou, Jiangsu), CoreValve, and Evolut R (Medtronic, Minneapolis, MN). Balloon-expandable valve choices included Prizvalve (NewMed Medical, Shanghai, China), Edwards SAPIEN 3, and SAPIEN XT (Edwards Lifesciences, Irvine, CA).

All ECGs were acquired using the ZONCARE iMAC 120 ECG machine (Wuhan Zoncare Bio-medical Electronics Co., Ltd) at a sampling rate of 500 Hz. Each ECG recording consisted of the traditional 12 leads recorded for 5 s, along with a 10-s trace of lead II. Other clinical and procedural information were obtained from the prospectively designed West China Hospital of Sichuan University Transcatheter Aortic Valve Replacement Registry (WATCH, ChiCTR2000033419). All of our TAVR patients were scheduled for a clinical follow-up at 30 days, 3 months, 6 months, 1 year, and yearly thereafter. Patients were contacted via phone to complete required tests, including an ECG test, in our hospital or local clinic. The study protocol was in accordance with the Declaration of Helsinki and approved by the Ethics Committee on Biomedical Research of West China Hospital (IRB number:2020470; date of IRB approval: 27 May 2020). All patients provided written informed consent.

### Endpoint definition and study group classification

The primary endpoint of the study was the occurrence of conduction disturbances within 30 days after TAVR, which was defined as the composite events of post-procedural PPMI, high-grade atrioventricular block (HAVB), and new-onset LBBB. The definitions of HAVB and LBBB were based on the AHA/ACCF/HRS criteria.^31,32^ Specifically, the HAVB includes the second-degree Mobitz type II atrioventricular block and the third-degree atrioventricular block. The multidisciplinary heart team was responsible for adjudicating the events and determining the need for PPMI based on 24-h Holter monitor or serial 12-lead ECGs after the procedure. Typically, persistent HAVB would trigger the need for PPMI, and patients with paroxysmal HAVB or progressive first-degree AVB (AVB I) with LBBB would be recommended for prophylactic PPMI. Any late-onset conduction disturbances would be assessed by the same team during the first follow-up visit at 30 days.

### Electrocardiogram selection and data pre-processing

We retrieved all ECGs taken during the hospitalization period before TAVR for patients who had multiple ECGs. This time frame was crucial for evaluating the indications for TAVR and making detailed operative plans. We randomly split the whole population into training and testing cohort on patient level to avoid data leakage. In the cross-validation set, all ECGs within this time frame were included to increase the sample size. In the hold-out testing set, only the last ECG before the procedure was included to simulate real clinical scenarios. We also performed a sensitivity analysis with a randomly selected ECG from this time frame in the testing.

To pre-process the original ECG images, we used the OpenCV Library to remove the background red grids and other noises. We standardized the pixel value of all ECG figures as 128 × 128 to facilitate subsequent training.

The missingness of clinical variables was scarce in our data set, with only seven patients presented with one or two missing values. Thus, we applied the simple imputation methods, substituting missing values with the mode for categorical variables and the mean for continuous variables. To address the imbalanced data, we used the imbalanced data set sampler for PyTorch to resample the data set.

### Overview of the network

We adopted the CNN as the main architecture of our network, implemented using PyTorch 1.5.0 and Python 3.7. CNN^16–18^ is a type of deep learning model specifically designed for tasks involving visual data. It employs layers of convolutional and pooling operations to automatically learn hierarchical features from input images, making it highly effective in recognizing patterns and structures within images. In this study, we developed two models to predict conduction disturbances following TAVR. The first model was solely based on ECG image data and was structured into two parts: the CNN part, which was responsible for extracting ECG image features, and the fully connected part, which was used for classification. The CNN part consisted of three basic blocks, each composed of two convolutional layers and one max-pooling layer. Each convolution layer had *n* filters shaped as 3 × 3 and was followed by a batch normalization layer to normalize data distribution and a ReLU activation function to generate non-linear representations. After the last CNN block, the data were fed into the fully connected network, which had two hidden layers, each followed by a ReLU activation function and a dropout layer to prevent overfitting. The output layer was activated by a Softmax function to achieve binary classification.

**Figure 1:**
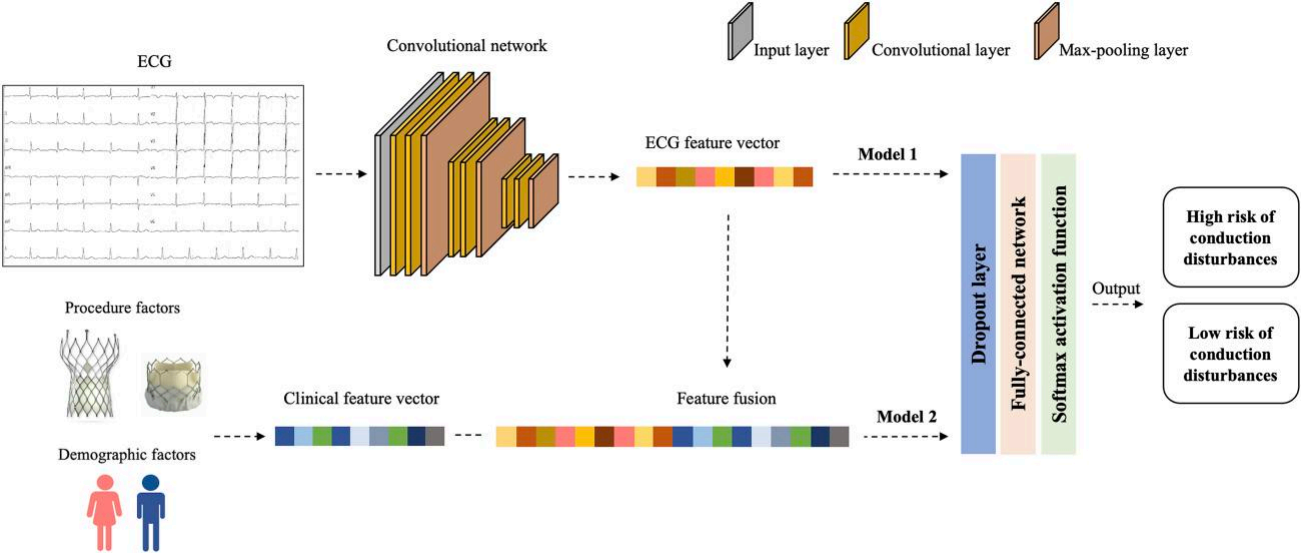
Architecture of the network. Two models were developed for predicting conduction disturbances after transcatheter aortic valve replacement. The first model utilized electrocardiogram data exclusively and comprised two components: a convolutional component for extracting features from the electrocardiogram image and a fully connected component for classification. The second model incorporated multimodal data. Clinical features were encoded as a clinical feature vector and combined with the ECG feature vector obtained from the convolutional component. The fused feature vector was then input into the fully connected classifier. ECG, electrocardiogram.

The second model incorporated multimodality data, specifically ECG images and clinical features. To combine image features with clinical features, we added a feature fusion part between the CNN part and the fully connected part. In this part, clinical features were encoded into a vector and concatenated with the image feature vector extracted by the CNN part. The fused feature vector was then fed into the fully connected classifier. The full architecture of our work is shown in *Figure 1*.

In this study, we applied ensemble learning techniques to enhance the generalization capabilities of neural networks. We started by using a five-fold cross-validation to train five sub-models on the cross-validation set. Next, we employed a voting-based method to combine these five sub-models into a unified final model. This final model was then employed to assess performance on a hold-out testing set. To optimize the network during training, we employed the Adam optimizer with focal loss function. This modified version of the cross-entropy loss function reduces the weight of easily classified samples and prioritizes the difficult ones during training. The training process was stopped once the best performance on the internal validation set was achieved, with hyperparameters fine-tuned to achieve the lowest loss value.

### Model comparisons and evaluations

For benchmark comparisons, we built three logistic regression models and three XGBoost^33^ models. These models were built using the following features: (i) high-risk pre-procedural ECG patterns (AVB I, RBBB), (ii) clinical features (age, sex, body mass index, pre-balloon dilation, valve type, valve size, and post-balloon dilation), and (iii) both ECG patterns and clinical features. The logistic regression models were trained utilizing the LogisticRegression function from the ‘sklearn’ Python package. And the XGBoost models were developed using the ‘xgboost’ Python package. All of these models were trained on the cross-validation set and validated on the hold-out testing set, following the training and testing steps of the CNN models. We also compared our model’s performance with the Emory score, a four-item scoring system developed by Kiani *et al.*^13^ to predict PPMI after TAVR.

We evaluated the models using the area under the curve (AUC) of the receiver operating characteristic (ROC) curve, accuracy, specificity, sensitivity, F1 score, negative predictive value (NPV), and positive predictive value (PPV).

### Testing and sensitivity analysis

The hold-out testing set was randomly selected with a 20:80 ratio from the entire sample at patient level to assess the transportability of the CNN models. The probability threshold used in the hold-out testing set was based on the AUC of the cross-validation set. The five sub-models built during cross-validation were integrated through voting ensemble technique to yield the ultimate model and then tested on the hold-out testing set. Only the most recent ECG taken before TAVR was included in the primary analysis. For sensitivity analysis, a random pre-TAVR ECG for each patient was selected, and this process was repeated five times.

### Statistical specifications

Continuous data were presented as mean ± SD and compared using Student’s *t* test. Categorical data were presented as numbers (percentages) and compared using the *χ*^2^ test or Fisher’s exact test. Two-tailed *P*-values were reported. Models were evaluated by AUC, accuracy, specificity, sensitivity, F1 score, NPV, and PPV with two-sided 95% confidence interval (CI) summarizing variability. The AUC results were compared using the Delong test^34^ for significance. Net reclassification improvement (NRI) was used to evaluate incremental predictive capability of the model.^35^ All statistical analyses were performed using R version 1.4.1717 and the Python package ‘sklearn’.

## Results

### Study flow and sample characteristics

**Figure 2:**
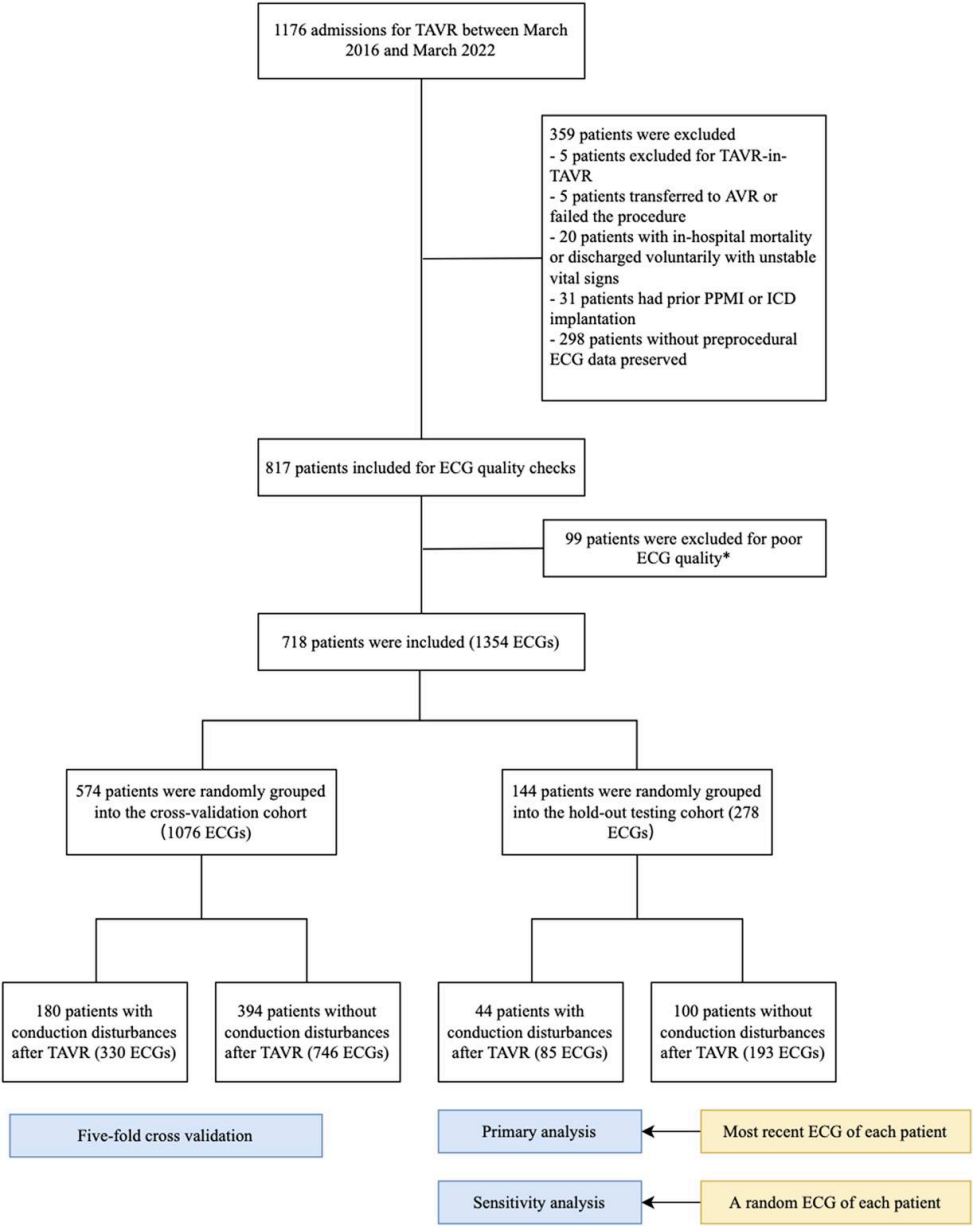
Study flowchart. In the study, a total of 1354 pre-procedural 12-lead electrocardiograms obtained from 718 patients were included. Out of these, a testing cohort comprising 278 electrocardiograms from 144 patients was hold out for testing. The models were trained and internally validated using a five-fold cross-validation method and subsequently tested on the hold-out testing set. The primary analysis was conducted using the most recent electrocardiogram obtained from each patient, while a sensitivity analysis was performed using a randomly selected electrocardiogram from each patient. *Examples of such quality issues include artefacts in the electrocardiogram, resulting from loosened electrodes or patient movement; stains on the electrocardiogram image that obscure or overlay the waveform; and indistinguishable waveforms on electrocardiogram image due to insufficient ink in the electrocardiogram machine. AVR, aortic valve replacement; ECG, electrocardiogram; ICD, implanted cardiac defibrillator; PPMI, permanent pacemaker implantation; TAVR, transcatheter aortic valve replacement.

A total of 1176 patients admitted for TAVR procedure between March 2016 and March 2022 to West China Hospital were retrospectively recruited. After applying the exclusion criteria, 817 patients were included in the study, and their ECGs were retrieved for quality checks. Of the remaining patients, 99 were excluded due to poor ECG quality. Examples of such quality issues include artefacts in the ECG, resulting from loosened electrodes or patient movement; stains on the ECG image that obscure or overlay the waveform; and indistinguishable waveforms on ECG image due to insufficient ink in the ECG machine. Eventually, a total of 1354 ECGs, obtained during hospitalization before TAVR, were included in the analysis from 718 patients. The hold-out testing set was composed of 144 patients, selected randomly with a 20:80 ratio from the entire sample (*Figure 2*). In the cross-validation cohort, 180 events (31.36%) were reported, comprising 135 (23.52%) cases of PPMI, 13 (2.26%) cases of new-onset LBBB with progressive AVB I, 23 (4.01%) cases of new-onset LBBB alone, and 9 (1.57%) cases of paroxysmal high-degree AVB. In the hold-out testing cohort, 44 events (30.56%) were observed, including 36 (25.00%) cases of PPMI, 4 (2.78%) cases of new-onset LBBB with progressive AVB I, 3 (2.08%) cases of new-onset LBBB alone, and 1 (0.69%) case of paroxysmal HAVB.

The baseline characteristics of the cross-validation set and hold-out testing set are presented in *Table 1* and were found to be balanced. The mean age was 72.8 and 73.0, respectively, and the proportion of female patients was 41.8 and 40.3%, respectively. Additionally, similar Society of Thoracic Surgeons (STS) scores and New York Heart Association (NYHA) classes were observed. With respect to procedural aspects, self-expanding valves were implanted in 91.4% of patients in the cross-validation set with 29.6% receiving prosthetic valve smaller or equal to size 23, while 93.1% of patients in the hold-out testing set received self-expanding valves with 29.9% receiving prosthetic valve smaller or equal to size 23. Baseline and procedural characteristics stratified by the occurrence of conduction disturbances are found in Supplementary material online, *Table S1*.

**Table 1:** Baseline characteristics of the cross-validation and hold-out testing cohort

	Cross-validation cohort (*n* = 574)	Hold-out testing cohort (*n* = 144)	*P*-value
Baseline characteristics			
Female sex	240 (41.8%)	58 (40.3%)	0.811
Age	72.76 ± 7.89	73.01 ± 7.62	0.740
Height, m	1.60 ± 0.08	1.60 ± 0.08	0.909
Weight, kg	57.85 ± 10.92	58.72 ± 10.00	0.391
BMI	22.70 ± 3.61	23.02 ± 3.33	0.335
STS score	3.78 ± 3.20	4.09 ± 3.30	0.315
NYHA class			0.787
I	9 (1.6%)	1 (0.7%)	
II	125 (22.6%)	33 (24.1%)	
III	344 (62.2%)	82 (59.9%)	
IV	75 (13.6%)	21 (15.3%)	
Hypertension	247 (43%)	60 (41.7%)	0.840
Diabetes mellitus	100 (17.4%)	29 (20.1%)	0.523
Chronic kidney disease	36 (6.3%)	7 (4.9%)	0.659
Chronic pulmonary disease	128 (22.3%)	37 (25.7%)	0.450
Coronary artery disease	125 (21.8%)	41 (28.5%)	0.111
Atrial fibrillation	86 (15.0%)	23 (16.0%)	0.925
Syncope	55 (9.6%)	14 (9.7%)	0.999
Native valve type			0.855
BAV	209 (36.4%)	50 (34.7%)	
Type 0	86 (15.0%)	20 (13.9%)	
Type 1	107 (18.6%)	28 (19.4%)	
Type 2	9 (1.6%)	0 (0.0%)	
TAV	359 (62.5%)	93 (64.6%)	
CrCl, mL/(min·1.73 mm^2^)	52.91 ± 19.96	53.75 ± 16.21	0.638
Echocardiographic characteristics			
AV maximal velocity, m/s	4.57 ± 0.98	4.56 ± 0.83	0.897
AV gradient, mmHg	54.60 ± 21.14	53.53 ± 17.75	0.600
LV diameter, mm	53.90 ± 10.06	53.42 ± 9.25	0.608
LV ejection fraction, %	56.09 ± 14.44	55.86 ± 14.26	0.864
Electrocardiographic characteristics			
AVB I	47 (8.2%)	12 (8.3%)	0.999
LBBB	53 (9.2%)	16 (11.1%)	0.599
RBBB	20 (3.5%)	6 (4.2%)	0.887
LAH	11 (1.9%)	2 (1.4%)	0.940
LPH	1 (0.2%)	0 (0.0%)	0.999
Procedural characteristics			
Pre-dilation	493 (85.9%)	120 (83.3%)	0.520
Prosthesis valve type			0.391
BE valve	49 (8.6%)	10 (6.9%)	
SE valve	522 (91.4%)	134 (93.1%)	
Prosthesis valve size ≤ 23	170 (29.6%)	43 (29.9%)	0.954
Prosthesis oversizing ≥ 16%	290 (50.5%)	73 (50.7%)	0.999
Post-dilation	235 (40.9%)	60 (41.7%)	0.949

Values are mean ± SD or frequency (percentage).

AV, aortic valve; AVB I, first-degree atrioventricular block; BAV, bicuspid aortic valve; BE, balloon-expandable; BMI, body mass index; CrCl, creatinine clearance; LAH, left anterior hemiblock; LBBB, left bundle branch block; LPH, left posterior hemiblock; LV, left ventricular; NYHA, New York Heart Association; RBBB, right bundle branch block; SE, self-expanding; STS, Society of Thoracic Surgeons; TAV, tricuspid aortic valve.

### Model performance on internal validation

**Figure 3:**
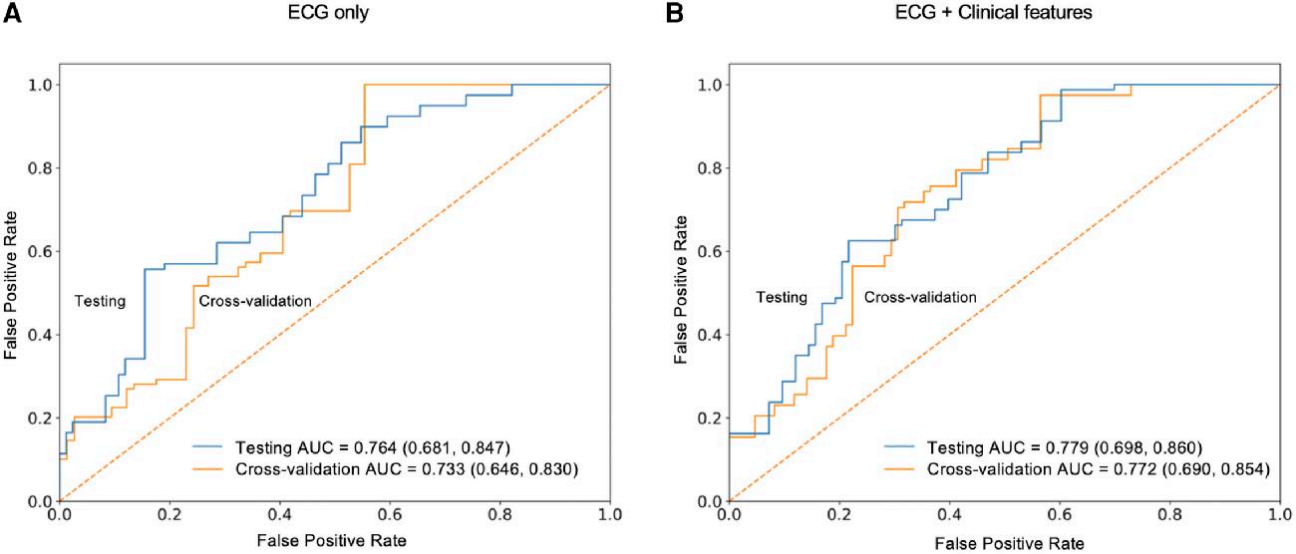
Receiver operating characteristics curve. (*A*) Curves for the model that used electrocardiogram only. (*B*) Curves for the enhanced model with additional clinical features.

The results of the CNN models’ performance on the five-fold cross-validation set are presented in *Table 2*, *Table S2* (*see Supplementary material online*) and *Figure 3*. Using the pre-TAVR ECG alone, the model showed an average AUC of 0.733, accuracy of 0.730, F1 score of 0.754, sensitivity of 0.851, specificity of 0.626, NPV of 0.849, and PPV of 0.678 for predicting conduction disturbances. We further investigated the effects of adding clinical characteristics to the CNN models. The results showed that there is an overall improvement of the performance with an AUC of 0.772, accuracy of 0.769, F1 score of 0.783, sensitivity of 0.818, specificity of 0.721, NPV of 0.796, and PPV of 0.746.

**Table 2:** Performance of the convolutional neural network models

	ECG image only		ECG image + clinical features	
	Cross-validation	Testing	Cross-validation	Testing
Sensitivity	0.851 (0.781, 0.921)	0.876 (0.811, 0.941)	0.818 (0.742, 0.894)	0.794 (0.715, 0.873)
Specificity	0.626 (0.531, 0.721)	0.624 (0.529, 0.719)	0.721 (0.633, 0.809)	0.752 (0.667, 0.837)
AUC	0.733 (0.646, 0.820)	0.764 (0.681, 0.847)	0.772 (0.690, 0.854)	0.779 (0.698, 0.860)
Accuracy	0.730 (0.643, 0.817)	0.743 (0.657, 0.829)	0.769 (0.686, 0.852)	0.774 (0.692, 0.856)
F1 score	0.754 (0.670, 0.838)	0.752 (0.667, 0.837)	0.783 (0.702, 0.864)	0.776 (0.694, 0.858)
PPV	0.678 (0.586, 0.770)	0.659 (0.566, 0.752)	0.746 (0.661, 0.831)	0.754 (0.670,0.838)
NPV	0.849 (0.779, 0.919)	0. 823 (0.748, 0.898)	0.796 (0.717, 0.875)	0.821 (0.746, 0.896)

Data are expressed as value (95% CI). In five-fold cross-validation, the average performance was presented. In testing cohort, the most recent ECGs before TAVR were used.

AUC, area under the curve; ECG, electrocardiogram; NPV, negative predictive value; PPV, positive predictive value.

### External validation and sensitivity analysis

The hold-out testing set comprising 278 ECGs of 144 patients was used to evaluate the performance of the CNN models. For the primary analysis, only the most recent ECG taken before TAVR of each patient was included. The results demonstrated consistent performance with the cross-validation set, with an AUC of 0.764, accuracy of 0.743, F1 score of 0.752, sensitivity of 0.876, specificity of 0.624, NPV of 0.823, and PPV of 0.659 (*Table 2* and *Figure 3*). To assess the impact of ECG image selection on model performance, a sensitivity analysis was performed by randomly selecting a pre-TAVR ECG of each patient, repeated five times. The average AUC was 0.769, with an accuracy at 0.735, F1 score at 0.758, sensitivity at 0.868, and specificity at 0.632 (see Supplementary material online, *Table S3*). We also performed a sub-group analysis exclusively targeting on patients receiving self-expanding valve, which showed stable performance with an AUC of 0.760. In another sub-group analysis on cohort without pre-existing LBBB, the model shows an AUC of 0.787.

The second model, which combined ECG images and clinical features, was also evaluated on the hold-out testing set, with an AUC of 0.779, accuracy of 0.774, F1 score of 0.776, sensitivity of 0.794, specificity of 0.752, NPV of 0.821, and PPV of 0.754, consistent with the performance in five-fold cross-validation (*Figure 3*) and demonstrating the model’s robustness.

### Model comparisons

In benchmark comparisons, we built three logistic regression models and three XGBoost models with (i) pre-existing RBBB and AVB I, (ii) clinical characteristics, and (iii) pre-existing RBBB, AVB I, and clinical characteristics. *Table 3* presents the AUCs of these models on the hold-out testing set. The results showed that the CNN models outperformed the logistic regression models and XGBoost models in predicting conduction disturbances after TAVR.

**Table 3:** Comparisons with logistic regression and XGBoost models

Models and input data	AUC	*P*-value^a^
CNN models		
ECG	0.764	—
ECG + clinical features	0.779	0.794
Logistic regression models		
Clinical features	0.637	0.060
High-risk ECG patterns	0.574	<0.001
High-risk ECG patterns + clinical features	0.675	0.170
XGBoost models		
Clinical features	0.528	<0.001
High-risk ECG patterns	0.520	<0.001
High-risk ECG patterns + clinical features	0.560	0.001

Clinical features include age, sex, body mass index, pre-balloon dilation, valve type, valve size, and post-balloon dilation. High-risk ECG patterns include first-degree atrioventricular block and right left bundle branch block.

AUC, area under the curve; CNN, convolutional neural network; ECG, electrocardiogram.

^a^Compared with CNN model trained with ECG data exclusively on the testing cohort.

Then, we compared the CNN model with the Emory score, a four-item scoring system developed by Kiani *et al.* to predict PPMI after TAVR. The CNN model demonstrated advantage over the Emory score with an AUC of 0.764 (95% CI: 0.681–0.847) and NRI of 0.729 (*P* < 0.0001) in the prediction of conduction disturbance (Emory score: AUC = 0.704, 95% CI: 0.623–0.785) and an AUC of 0.730 (95% CI: 0.643–0.817) and NRI of 0.661 (*P* = 0.0007) in the prediction of PPMI (Emory score: AUC = 0.679, 95% CI: 0.58–0.776, *P* = 0.160) in the hold-out testing cohort.

## Discussion

This study developed an AI model that effectively predicts conduction disturbances after TAVR using pre-procedural 12-lead ECG images. The performance of this AI tool was found to be comparable to existing models that predict pacemaker implantation after TAVR, with an AUC of 0.733 in the cross-validation set and 0.764 in the hold-out testing set. The performance of this AI model was superior to logistic and XGBoost models built with manually defined high-risk ECG patterns, as well as the Emory score. To the best of our knowledge, this is the first study that used AI-enhanced ECG to predict conduction disturbance concerning TAVR.

Both PPMI and new-onset LBBB can have a negative impact on morbidity and mortality following TAVR. While conflicting data exist regarding the effect of PPMI on mortality, recent population-based analyses^36,37^ have demonstrated an association between PPMI and increased rates of all-cause mortality and heart failure hospitalization. New-onset LBBB typically occurs during manipulation of the aortic root or immediately after the procedure and is linked with an increased risk of 1-year PPMI, as well as reduced LVEF recovery and increased rates of all-cause mortality, cardiovascular mortality, and heart failure hospitalization.^36,38^ These risks should be given special attention since TAVR is increasingly being used in younger and healthier population. Patients should be informed of these risks and the long-term implications, as well as the associated extra cost,^39^ before deciding on the most appropriate procedure. Operators can also minimize these risks by adopting coping strategies such as using balloon-expandable valves, avoiding unnecessary balloon dilation, and restricting prosthesis oversizing.

In this study, we utilized modern computational technology to evaluate a widely used, low-cost, and easily accessible clinical test. Previous studies aiming to predict conduction disturbances after TAVR have often relied on pre-procedural ECG features, including RBBB, QRS duration, LBBB, AVB, and bradycardia.^12,13,15^ However, these traditional methods have limitations. Firstly, while the ECG test itself is objective, human interpretation can introduce subjective factors affected by the interpreter’s level of experience and expertise,^40^ as well as their understanding of the patient’s condition. Secondly, even if automatic interpretations were adopted, they were based on human-defined rules that may not capture all the intricate information in ECGs. In contrast, deep learning algorithms learn information and relationships in an outcome-directed way, independent of predefined rules. We trained our network on original ECG images, using corresponding labels (outcomes), to adjust the weight of each parameter until the predicted outcomes were most proximate to the actual outcome.^40^ Additionally, CNN algorithms exhibit greater flexibility in handling non-linear relationships and interactions than traditional statistical models. The superior performance of CNN models over logistic regression, XGBoost models, and Emory score in our study may be attributed to these factors.

Studies on AI-enabled ECG analysis primarily use either 1D ECG signals or 2D ECG images. Although most large ECG databases utilized for developing AI models consist of ECG signal data, studies using ECG images have also been published.^28^ Convolutional neural networks were originally designed to handle computer vision tasks and are now widely applied in medical image analysis,^41^ making ECG images an ideal substrate for CNN network training. Especially, Sangha *et al.*^42^ highlighted that models trained with ECG images are non-inferior to those trained with raw signals while offering broader applicability in various healthcare settings without being restricted by storage strategies.

In our second model, we added several basic clinical features, including four procedural features. These features were selected based on their evidence-proven association, and they are explicit core variables that can be easily retrieved from almost every TAVR centre. This enhanced model was developed for TAVR operation planning and postoperative evaluation. For high-risk patients during postoperative evaluation, close ECG monitoring could be performed, and if necessary, early PPMI could be considered to reduce the length of hospital stay.

In contrast to existing risk scores, such as the Emory risk score and the risk score developed by Maeno *et al.*, which are specifically tailored to recipients of the balloon-expandable Edwards SAPIEN 3 valve, our model had no restrictions on valve type and brand. However, predictions pertaining to patients scheduled to receive balloon-expandable valve could be overestimated and, therefore, should be approached with caution, given that recipients of balloon-expandable valves constitute only 10% of the cohort. An AI model that incorporates two distinct sub-networks to deal with the two different populations is to be developed given sufficient sample size in future research, thereby providing a targeted and potentially more accurate prediction for each valve modality.

The main advantage of this model lies in its simplicity, accessibility, and generalizability. The model was built on a real-world population that encompass a comprehensive spectrum of patients. The model demonstrated high sensitivity and NPVs, and it can provide rapid prediction results within seconds, making it cost-effective screening tools, particularly for providing reassurance to low-risk patients. However, it has to be noted that these predictions are not mean to serve as decision maker, rather, they should be taken as one side of the evidence to aid clinicians make informed decisions about the choice between AVR and TAVR, the selection of prosthesis valve devices, and the need for prophylactic pacing or close monitoring.

Several limitations exist regarding our present work. Firstly, this is a single-centre study with a relatively limited sample size compared with existing studies on AI-enabled ECGs. However, given our target population, which comprises recipients of a novel intervention introduced in the last two decades, our sample size is acceptable as a preliminary study. Continuous calibration with more samples from different centres is required to optimize the model. Secondly, our work is retrospective in nature, resulting in the exclusion of numerous patients with ineligible ECG images. Nonetheless, we fully utilized all available data with oversampling techniques and evaluated our model in the hold-out testing cohort. Finally, our model lacks external validation with data from other centres.

## Conclusions

In this study, we developed and validated an AI model that demonstrated good performance in predicting conduction disturbances after TAVR using pre-procedural 12-lead ECG images, with incremental performance observed when clinical variables added. Our results indicate that AI-enhanced ECG images may offer better predictive value than traditionally defined high-risk ECG patterns in the TAVR setting. This AI tool can potentially provide valuable information for shared decision-making regarding the most appropriate procedure to treat aortic stenosis and to aid in TAVR operative planning.

## Supplementary Material

ztae007_Supplementary_Data

## Data Availability

The data that support the findings of this study are available from the corresponding authors upon reasonable request and with permission of the West China Hospital. Analysing code could be found at the following link: https://github.com/gaden168/pytorch-ecg.git.
