# Supplementary material for: Electrocardiogram-based prediction of conduction disturbances after transcatheter aortic valve replacement with convolutional neural network
Source: Eur Heart J Digit Health. 2024 Feb 8;5(3):219–28. doi: 10.1093/ehjdh/ztae007 (PMC11104474; doi:10.1093/ehjdh/ztae007)
Supplement: ztae007_Supplementary_Data [file ztae007_supplementary_data.docx]

**Supplementary Materials**

[Supplementary Table 1: Baseline characteristics according to the occurrence of conduction disturbances after TAVR. 2](#_Toc129773257)

[Supplementary Table 2: Individual results of 5-fold cross validation 4](#_Toc129773258)

[Supplementary Table 3: Sensitivity analysis of the CNN model. 4](#_Toc129773259)

# Supplementary Table 1: Baseline characteristics according to the occurrence of conduction disturbances after TAVR.

|  | **No Conduction Disturbances (n=494)** | **Conduction Disturbances (n=224)** | **p value** |
| --- | --- | --- | --- |
| **Baseline characteristics** | | |  |
| Female sex | 220 (44.5%) | 78 (34.8) | 0.018 |
| Age | 72.17 ± 8.18 | 74.24 ± 6.79 | 0.001 |
| Height, m | 1.59 ± 0.08 | 1.61 ± 0.08 | 0.015 |
| Weight, kg | 57.17 ± 10.57 | 59.92 ± 10.90 | 0.002 |
| BMI | 22.55 ± 3.56 | 23.24 ± 3.50 | 0.016 |
| STS score | 3.74 ± 3.09 | 4.07 ± 3.49 | 0.221 |
| NYHA class |  |  | 0.018 |
| I | 5 (1.0%) | 5 (2.3%) |  |
| II | 97 (20.3%) | 61 (28.6%) |  |
| III | 300 (62.9%) | 126 (59.2%) |  |
| IV | 75 (15.7%) | 21 (9.9%) |  |
| Hypertension | 188 (38.1%) | 119 (53.1%) | <0.001 |
| Diabetes mellitus | 82 (16.6%) | 47 (21.0%) | 0.189 |
| Chronic kidney disease | 27 (5.5%) | 16 (7.1%) | 0.479 |
| Chronic pulmonary disease | 109 (22.1%) | 56 (25%) | 0.441 |
| Coronary artery disease | 104 (21.1%) | 62 (27.7%) | 0.064 |
| Atrial fibrillation | 74 (15.0%) | 35 (15.6%) | 0.912 |
| History of syncope | 48 (9.7%) | 21 (9.4%) | 0.994 |
| Native valve type |  |  | 0.041 |
| BAV | 193 (39.1%) | 66 (29.5%) |  |
| Type-0 | 86 (17.4%) | 20 (8.9%) |  |
| Type-1 | 93 (18.8%) | 42 (18.8%) |  |
| Type-2 | 8 (1.6%) | 1 (0.4%) |  |
| TAV | 297 (60.1%) | 155 (69.2%) |  |
| CrCl, ml/(min·1.73mm^2) | 53.16 ± 19.27 | 52.89 ± 19.26 | 0.860 |
| **Echocardiographic characteristics** | | |  |
| AV maximal velocity, m/s | 4.66 ± 0.94 | 4.37 ± 0.94 | <0.001 |
| AV gradient, mmHg | 56.28 ± 20.76 | 50.09 ± 19.30 | <0.001 |
| LV diameter, mm | 53.78 ± 10.33 | 53.86 ± 8.91 | 0.920 |
| LV ejection fraction, % | 55.49 ± 15.25 | 57.27 ± 12.22 | 0.131 |
| **Electrocardiographic characteristics** | | | |
| AVB I | 33 (6.7%) | 26 (11.6%) | 0.033 |
| LBBB | 36 (7.3%) | 33 (14.7%) | 0.003 |
| RBBB | 11 (2.2%) | 15 (6.7%) | 0.006 |
| LAH | 11 (2.2%) | 2 (0.9%) | 0.347 |
| LPH | 1 (0.2%) | 0 (0.0%) | 0.999 |
| **Procedural characteristics** | | |  |
| Pre-dilation | 430 (87.0%) | 183 (81.7%) | 0.078 |
| Prosthesis valve type |  |  | 0.094 |
| BE | 48 (9.7%) | 11 (4.9%) |  |
| SE | 444 (89.9%) | 212 (94.6%) |  |
| Prosthesis valve size | 25.94 ± 2.82 | 26.65 ± 2.47 | 0.001 |
| Prosthesis Oversizing ≥ 16% | 236 (47.8%) | 127 (56.7%) | 0.033 |
| Post-dilation | 202 (40.9%) | 93 (41.5%) | 0.939 |
| Values are mean ± SD or frequency (percentage). AV = aortic valve; AVB I = first degree atrioventricular block; BAV = bicuspid aortic valve; BE = balloon-expandable; BMI = body mass index; CrCl = Creatinine clearance; LAH = left anterior hemiblock; LBBB = left bundle branch block; LBBB = left bundle branch block; LV = left ventricular; NYHA = New York Heart Association; RBBB = right bundle branch block; SE = self-expanding; STS = Society of Thoracic Surgeons; TAV = tricuspid aortic valve. | | | |

# Supplementary Table 2: Individual results of five-fold cross-validation

|  | **Sensitivity** | **Specificity** | **AUC** | **F1** | **Accuracy** |
| --- | --- | --- | --- | --- | --- |
| Fold 1 | 0.704 | 0.693 | 0.708 | 0.727 | 0.715 |
| Fold 2 | 0.970 | 0.526 | 0.765 | 0.787 | 0.767 |
| Fold 3 | 0.652 | 0.643 | 0.676 | 0.690 | 0.687 |
| Fold 4 | 0.964 | 0.628 | 0.754 | 0.779 | 0.744 |
| Fold 5 | 0.966 | 0.642 | 0.763 | 0.785 | 0.737 |
| **Average** | 0.851 | 0.626 | 0.733 | 0.754 | 0.730 |

# Supplementary Table 3: Sensitivity analysis of the CNN model.

| **Random selection** | **Sensitivity** | **Specificity** | **AUC** | **F1** | **Accuracy** |
| --- | --- | --- | --- | --- | --- |
| Round 1 | 0.881 | 0.673 | 0.789 | 0.779 | 0.765 |
| Round 2 | 0.813 | 0.630 | 0.748 | 0.727 | 0.727 |
| Round 3 | 0.894 | 0.611 | 0.776 | 0.766 | 0.742 |
| Round 4 | 0.846 | 0.675 | 0.752 | 0.747 | 0.698 |
| Round 5 | 0.904 | 0.572 | 0.779 | 0.772 | 0.741 |
| **Average** | 0.868 | 0.632 | 0.769 | 0.758 | 0.735 |
| To test whether the selection of ECG images affect model's performance, we performed a sensitivity analysis by randomly selecting a pre-TAVR ECG of each patient. This process was repeated for five rounds. | | | | | |
